# Autophagy: Regulator of cell death

**DOI:** 10.1038/s41419-023-06154-8

**Published:** 2023-10-04

**Authors:** ShiZuo Liu, ShuaiJie Yao, Huan Yang, ShuaiJie Liu, YanJiao Wang

**Affiliations:** 1 School of Basic Medical Sciences, Xinjiang Medical University, Urumqi, China; 2 The Second School of Clinical Medicine, Xinjiang Medical University, Urumqi, China; 3 Xinjiang Key Laboratory of Molecular Biology for Endemic Diseases, Department of Biochemistry and Molecular Biology, School of Basic Medical Sciences, Xinjiang Medical University, Urumqi, China

**Keywords:** Macroautophagy, Cancer metabolism

## Abstract

Autophagy is the process by which cells degrade and recycle proteins and organelles to maintain intracellular homeostasis. Generally, autophagy plays a protective role in cells, but disruption of autophagy mechanisms or excessive autophagic flux usually leads to cell death. Despite recent progress in the study of the regulation and underlying molecular mechanisms of autophagy, numerous questions remain to be answered. How does autophagy regulate cell death? What are the fine-tuned regulatory mechanisms underlying autophagy-dependent cell death (ADCD) and autophagy-mediated cell death (AMCD)? In this article, we highlight the different roles of autophagy in cell death and discuss six of the main autophagy-related cell death modalities, with a focus on the metabolic changes caused by excessive endoplasmic reticulum-phagy (ER-phagy)-induced cell death and the role of mitophagy in autophagy-mediated ferroptosis. Finally, we discuss autophagy enhancement in the treatment of diseases and offer a new perspective based on the use of autophagy for different functional conversions (including the conversion of autophagy and that of different autophagy-mediated cell death modalities) for the clinical treatment of tumors.

## Facts

- Autophagy is involved in the regulation of almost all modes of cell death in disease contexts.
- Autophagy plays a role in protecting cells against most diseases, and lethal autophagy is usually induced by pharmacological or genetic treatment.
- Excessive autophagy-induced cell death is caused by excessive degradation of cellular contents and involves various metabolic mechanisms and organelles.

## Open questions

- Protective autophagy and lethal autophagy may be simultaneously triggered in cells. How do cells balance the regulatory mechanisms of these processes?
- What is the biological mechanism explaining why cells undergo excessive autophagy?
- What is the role of autophagy in the phagocytosis of organelles in cells undergoing ferroptosis?
- Can controlling the conversion of autophagy function contribute to the treatment of cancer, neurodegenerative diseases, inflammation, cell senescence and cardiovascular diseases.

## Introduction

Death is the end of all living things. In recent decades, scientists have gradually unraveled the mystery of cell death [1]. Moreover, the roles of autophagy in cell death have received increasing attention, but the precise mechanisms underlying the roles are still unclear. Macroautophagy (hereafter referred to as autophagy) is a process by which cellular contents are encircled by the membrane-bound structures known as autophagic vesicles, which eventually fuse with lysosomes; in these vesicles, the cellular contents are broken down into small molecules [2]. Autophagy plays a dual role in various diseases. For example, in the early stages of tumorigenesis, autophagy plays a tumor-suppressing role by helping maintain genomic integrity and inhibiting tissue damage and inflammation through processes involving quality control systems and oxidative stress responses [3, 4]. However, in the advanced stage of tumor development, autophagy provides nutrients to cancer cells and promotes their immune escape (by promoting the degradation of MHC-I on the surface of cancer cells [5]), as well as other functions [2, 6]. The effects of autophagy on organ tissues appear to be related to the presence of disease or health of an organism [7]. Moreover, autophagy seems to determine whether a cell lives or dies [8–10]. The roles of autophagy in cell death are mainly classified into autophagy-dependent cell death (ADCD) (or autophagic cell death, ACD), which depends on the autophagy mechanism, and autophagy-mediated cell death (AMCD), which depends on other modes of cell death. In ADCD, the unrestricted degradation of cellular contents leads to a disrupted cellular environment; however, is the mechanism truly a simple case of excessive self-eating? In AMCD, autophagy drives different modes of death as the basis for the initiation of other death modes; however, does autophagy also regulate the switching to different modalities of cell death after clinical treatment? In this paper, we highlight the various forms of autophagy and the mechanisms that mediate cell death, explaining the shift in cells undergoing AMCD.

## Autophagosome formation

**Fig. 1:**
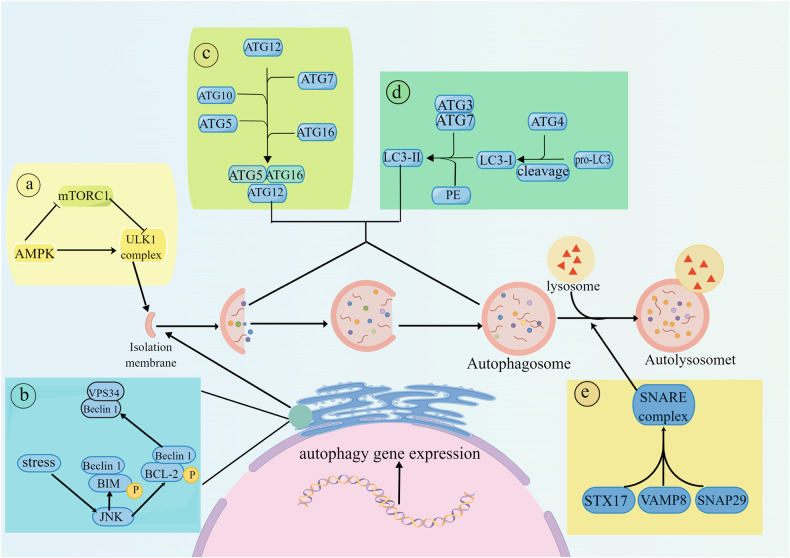
Mechanism of autophagy. Autophagy is a complex self-degradation process involving the following key steps: ①Signaling pathways regulate the initiation of autophagy. AMPK inhibits the formation of the mTORC1 complex, which weakens the inhibitory effect of mTORC1 on the formation of the ULK1 complex, thereby promoting the production of autophagic vesicles. ② The Beclin-1/VPS34 complex promotes the extension of autophagic vesicles. The activated kinase JNK destroys the Beclin1/BCL-2 and Beclin1/BIM complexes by phosphorylating BCL-2 and BIM to free Beclin1. Free Beclin1 activates VPS34 and binds to it to form a complex, and the PI3P that is produced promotes the extension of autophagic vesicles. ③ The ATG12-ATG5 complex binds to ATG16 and completes polymerization. The polymer complex ATG5-ATG12-ATG16L is formed by a series of ATG5, ATG12 and ATG16L actions, and then, the polymer complex is fused with autophagic vesicles. ④ LC3 is inserted into autophagosomes through a series of reactions. The cysteine protease ATG4 cleaves LC3 into LC3-I, which is then processed by ATG3, ATG7 and phosphatidylethanolamine to form LC3-II. Subsequently, LC3-II is inserted into the autophagosomes. ⑤ Autophagosomes and lysosomes fuse to form autolysosomes. STX17 binds to SNAP29 and VAMP8 to form a SNARE complex that is transferred to the autophagosomal membrane, allowing lysosomes to fuse with autophagosomes and form autolysosomes. The graph was drawn with Figdraw.

Since the discovery of the autophagy-related genes (ATGs) in brewer’s yeast by Yoshinori Ohsumi in the 1990s [11], scholars have identified more than 40 ATGs in yeast by genetic screening, and approximately one-half of these genes show clear homology to ATGs in mammals. AMPK and mTOR signaling initiate autophagy, and their function is mediated through cellular responses to stress, such as starvation or oxidation (Fig. 1). ULK1 Ser757 is dephosphorylated by protein phosphatase 2A (PP2A) [12] and protein phosphatase 1D magnesium-dependent delta isoform (PPM1D) [13] after the dissociation of mTOR from ULK1, and AMPK phosphorylates ULK1 Ser 317 and Ser 777 to promote the formation of the ULK1 complex [14]. OGT co-mediates the phosphorylation of ATG14 Ser29 with GABARAPs after the acetylation of ULK1 Ser757 by O-GlcNAc [15, 16]. After activation of the ULK1 complex, PIKfyve Ser1548 is phosphorylated to promote the conversion of PI to PI(5)P, with the latter subsequently binding WIPI2 to promote separation of the isolation membrane from the ER membrane [2, 17, 18]. To continue the extension of the isolation membrane, the PI3KC3 complex binds to the ATG5-ATG12 complex and the ATG8/LC3 system via WIPI2, promoting ATG5-ATG12 complex and ATG8/LC3 system ubiquitination via a positive feedback loop until the isolation membrane closes, forming an autophagosome [19, 20]. Then, autophagosomes recruit lysosomal fusion proteins, while ATGs on the outer autophagosome membrane are successively removed. STX17 is deacetylated, and C-terminal hairpin-like structures are inserted into intact autophagosomes, where they interact with SNAP29 and HOPS to facilitate autophagosome–lysosome binding [21, 22]. Ultimately, the cellular cargo is degraded into small molecules by the lysosomes and recycled.

## Autophagy and cell death

In addition to maintaining intracellular energy and metabolic homeostasis, autophagy can mediate cell death under certain conditions. The following two main types of autophagy-related cell death modes have been described: 1) ADCD, which is highly dependent on autophagy components and is independent of other forms of programmed death. For cell death to be attributed to ADCD, ① the autophagic flux must be elevated during cell death; ② the cell death process must be reversible via genetic or pharmacological inhibition of autophagy; ③ the death process depends on at least two autophagy molecules, which prevents individual molecules from mediating cell death independent of autophagy; and ④ the death process must not be accompanied by other modes of death [23, 24]. 2) In AMCD, autophagic molecules interact directly with cell death molecules or autophagy triggers apoptosis, necrosis, and ferroptosis via dynamic autophagy functions [8, 25]. The two forms of autophagy-related cell death are not independent of each other and can be triggered simultaneously, and cell death processes may switch between these two modalities under certain conditions [26]. Herein, we describe the six modes of cell death mediated by ADCD (endoplasmic reticulum-phagy [ER-phagy], mitophagy, and autosis) and AMCD (apoptosis, necroptosis, and ferroptosis).

## Autophagy-dependent cell death

ADCD is involved in the degradation of the larval midgut during *Drosophila* development, and knockdown of the associated ATG-encoding genes significantly delays midgut degradation [27]. In embryonic model mice with Bax/Bak knocked out, autophagy facilitated elimination of interdigital web cells. Autophagosomes appear to predominantly breakdown cell matrix components with no apparent commensurate degradation of organelles [28]. ADCD is important to epidermal keratin-forming cells in adult mammalian skin, and autophagy regulates terminal cell death by degrading organelles, such as the ER or mitochondria, or intracellular membranes [29]. In recent years, excessive autophagy in tumor or cardiovascular disease cells has been widely reported and classified into ER-phagy, excessive mitophagy, and autosis. These three types of autophagy are described in this section.

### Excessive ER-phagy

#### Introduction to excessive ER-phagy

The ER is the largest organelle in eukaryotic cells. It can form a complex cell quality control network by contacting other organelle membranes and participates in Ca^2+^ homeostasis and protein synthesis [30]. The ER activates protective mechanisms under stress conditions, such as a lack of nutrients or oxygen, including the unfolded protein response (UPR), ER-associated degradation (ERAD) and ER-phagy. Although ER-phagy was observed as early as 1965 [31], how the isolation membrane specifically recognizes the ER was not known until more recently. In 2015, FAM134B was the first ER-phagy receptor to be identified. In mammalian cells, FAM134B carries an LC3 interaction region (LIR), which specifically recognizes the LC3/GABARAP family, and an ER homology domain (RHD), which is involved in fragmenting the ER into small segments, enabling engulfment by the autophagosome [32]. In yeast, the FAM134B homologs ATG39 and ATG40 also regulate ER homeostasis via ER-phagy [33].

**Fig. 2:**
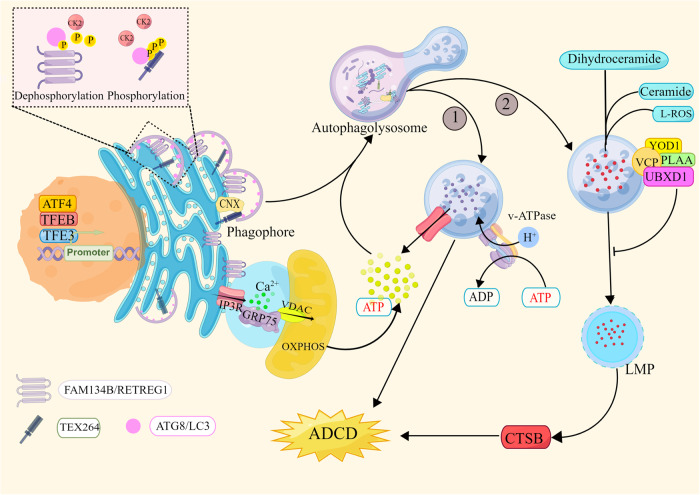
Excessive ER-phagy-mediated cell death. ① The transcription factors ATF4, TFEB, and TFE3 induce the expression of the ER-phagy receptors FAM134B/RETREG1 and TEX264 after pharmacological treatment, thereby initiating the ER-phagy program. Dephosphorylated CK2 phosphorylates the LIR in FAM134B and TEX264 to enhance their ability to bind LC3. The ER is cleaved into small fragments, which are wrapped by the isolation membrane and eventually degraded by lysosomes. Excessive ER consumption eventually leads to cell death. ②Excessive ER-phagy leads to the disruption of lipid and cholesterol metabolism, and lipid accumulation in lysosomes results in the accumulation of Lipid-ROS and the release of LMP, with CTSB leading to ADCD. The lysosomal receptor VCP and its cofactors YOD1, PLAA, and UBXD1 induce the degradation of damaged lysosomes to mitigate ADCD. OXPHOS provides energy for ER-phagy, and FAM134B protects mitochondria by inhibiting ROS, degrading CNX, and inhibiting Ca^2+^ conductance mediated by the IP3R-GRP75-VDAC complex. The graph was drawn with Figdraw.

The primary function of ER-phagy under stress condition is degradation of the expanded ER membrane to maintain cellular quality and balance [34]. The UPR produces upstream activation signaling that triggers autophagy [35], which activates ATF4, inositol-requiring enzyme 1 (IRE1), and other molecules to mediate ADCD after pharmacological treatment [36, 37]. Eleven ER-phagy receptors have been identified [34], and two of these proteins have been reported to be involved in ADCD. Loperamide treatment of glioblastoma (GBM) promotes the UPR, and downstream ATF4 mediates the activation of the ER-phagy receptors FAM134B and TEX264 to promote ER degradation [38]. ER function is impaired by the continuous degradation of ER fragments, eventually leading to cell death. However, some studies have described different mechanisms to explain ER-phagy. In stressed environments, FAM134B activity is upregulated to promote ER-phagy, continuously degrading the ER and impairing its function until the UPR is activated (Fig. 2) [39]. Although the study explaining this process did clearly explain the relationship between ER-phagy receptors, autophagy and the UPR, it is commonly thought that the UPR sends upstream activation signals to trigger autophagy and induce autophagy receptor expression [35, 38, 40], as is discussed below.

#### The energy context of excessive ER-phagy

Neither ER stress nor autophagy is mediated by a complex regulatory signaling network, and the cell metabolism context of excessive ER-phagic environments cannot be fully explained by UPR signaling, ER-phagy or ER damage. During excessive ER-phagy, approximately fivefold more autophagic vacuoles are produced than that which are produced in cells under physiological conditions, and twice as many as those in the stressed state [39]; twice as many autophagosomes implies doubled energy expenditures. After 30%-50% of ATP is consumed, the autophagosome volume decreases by 70% [41]. How do cells maintain constant autophagic flux when energy is constantly depleted? The functions of two receptors mediating ADCD, FAM134B and TEX264, reveal the answer [38, 39]. To attenuate the disruption to energy homeostasis in cells undergoing excessive autophagic flux, activation of the transcription factors TFEB and TFE3 promotes the expression of FAM134 family proteins and TEX264, and the regulation of casein kinase 2 (CK2) mediated via phosphorylation enhances the affinity of autophagy receptors for LC3 (Fig. 2). FAM134B and TEX264 at the ER lamellae and tubule junctions, respectively, are activated to mediate autophagosome envelopment of ER fragments and their further breakdown into small-molecule metabolites [42–45]. Thus, FAM134B and TEX264 mediate the degradation of more than 50% of ER fragments within 15 min [42, 46], effectively balancing the energy consumed via excessive autophagic flux. However, pharmacological treatments cause high stress levels in cells, and the energy recycled via autophagy alone is clearly not sufficient to sustain the energy expenditure associated with cell quality control and the repair of damaged proteins or DNA. Meyer et al. observed that mitochondria remained unexpectedly intact during excessive ER-phagy [47], suggesting that mitochondria may be regulated via protective mechanisms that enable them to function. A genome-wide CRISPR interference (CRISPRi) reporter gene screening with the HCT116 colorectal cancer cell line by Liang et al. revealed that the oxidative phosphorylation system (OXPHOS) stimulated ER-phagy through a nonclassical energy-regulating pathway [48]. The metabolism in tumor cells easily changes from aerobic glycolysis to OXPHOS through metabolic reprogramming [49], and this efficient metabolic shift addresses the energy balance discussed above. However, how do cells ensure that mitochondria safely undergo OXPHOS in a high-stress context? To ensure mitochondrial function, FAM134B reduces mitochondrial damage by inhibiting Ca^2+^ and reactive oxygen species (ROS) production. FAM134B inhibits Ca^2+^ hypo-transduction in the mitochondria-associated ER membrane (MAM) by the inositol-1,4,5-trisphosphate receptor (IP3R)-glucose regulatory protein 75 (Grp75)-voltage-dependent anion channel (VDAC) complex, which is involved in MAM formation. VDAC and IP3R are channels through which Ca^2+^ enters mitochondria. Excessive conduction of Ca^2+^ may lead to mitochondrial dysfunction, and FAM134B reduces Ca^2+^ transfer between MAMs by inhibiting IP3R activity, but the exact mechanism is not clear. In addition, FAM134B induces ER-phagy to directly degrade calmodulin and CNX and attenuates mitochondrial Ca^2+^ damage [50, 51]. Additionally, FAM134B overexpression mitigates ROS accumulation [52]. In contrast, although FAM134B has been suggested to interact with the mitochondrial inner membrane protein OPA1 and the FAM134B LIR motif plays a role in mitochondria degradation by assembling phagocytic factors around mitochondria [53], cells in models of excessive ER-phagy preferentially protect energy-producing structures [47].

#### Other mechanisms underlying excessive ER-phagy

Loperamide and pimozide induce excessive ER-phagy in patients with GBM and upregulation of cholesterol and lipid anabolism, leading to the accumulation of cholesterol and lipids (predominantly ceramide) in lysosomes [47]. The accumulation of lipids leads to lysosomal membrane permeabilization (LMP), which contributes to lipid-ROS production and cathepsin B (CTSB) release [47]. Moreover, if a rapid lysosomal repair mechanism mediated by phosphoinositide and the ER does not function properly due to high Lipid-ROS production rates and the continuous depletion of ER function [54], this can ultimately lead to autophagy-mediated LMP-related death. Interestingly, p97/VAP is translocated to the surface of lysosomes after cells are damaged and recruits cofactors YOD1, UBXD1, and PLAA to induce lysophagy, through which the damaged lysosomes are degraded, promoting GBA cell survival (Fig. 2) [47, 55]. This outcome indicates that protective and lethal autophagy may both be triggered and balanced and that triggering the activation of certain molecules may lead to cell death mediated by an imbalance in autophagy.

The membrane source of excessive ER-phagy is unknown and an interesting area of inquiry. Isolation membranes usually form at the ER, Golgi apparatus or MAM [18], and different membrane sources may accelerate excessive ER-phagy-mediated ADCD. When the predominant membrane source is the ER, ER-phagy promotes ER membrane consumption, and isolation membrane formation accelerates this process. Interestingly, FAM134B knockdown reduced the volume of the Golgi apparatus by 40% without altering the cell volume [56], suggesting that FAM134B may influence the source of membranes of autophagosomes by regulating the volume of organelles. In conclusion, the dynamic structure of the ER dictates that its size needs to be coordinated with that of other organelles to maintain physiological cell functions [30], and further investigation into the fine-tuned regulatory mechanisms among organelles in the context of excessive ER-phagy may facilitate the development of new ADCD-related therapeutic strategies.

### Mitophagy

Mitochondria are involved in almost all critical steps in tumor development, including the production of ATP and ROS, functioning as hubs for anabolism and regulated cell death (RCD) signaling [57]. Mitochondria have complex quality-regulatory mechanisms, which primarily balance the removal of damaged or depolarized mitochondria and mitochondria biogenesis, and excessive mitophagy leads to defects in mitochondrial function and cell death.

Mitophagy-triggered ADCD was first identified in 2006, when Reef et al. found that overexpression of an alternative translation product of ARF (p19), namely, short mitochondrial ARF (smARF), led to an abnormal mitochondrial membrane potential and promoted mitophagy-mediated ADCD [58]. Budina-Kolomets et al. further found that full-length ARF induced autophagy, but smARF bound Parkin to induce mitophagy [59, 60].

**Fig. 3:**
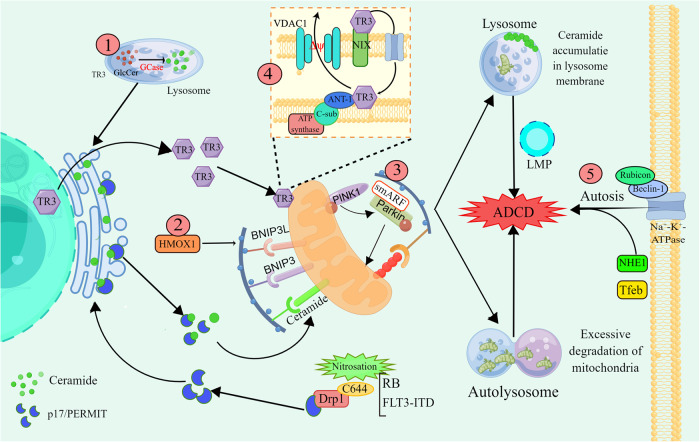
Mechanisms underlying lethal mitophagy and autosis. ① GCase overexpression promotes lysosomal glucose ceramide catabolism into glucose and lysosomal ceramide, which are recycled in the ER. Under conditions of RB or FLT3-ITD inhibition, Drp1 C644 nitrosylation leads to the release of the transport protein p17/PERMIT. Ceramide is transported from the ER to mitochondria by p17/PERMIT, which functions as a mitophagy receptor, and its binding to lipidated LC3 promotes lethal mitophagy. In addition, ceramide or dihydroceramide accumulates in autophagosomal membranes to trigger LMP. ②AT-101 induces the transcription and translation of HMOX1, BNIP3L and BNIP3 and promotes excessive mitophagy. ③smARF promotes excessive mitophagy by binding to Parkin. ④ TR3 is translocated from the nucleus to mitochondria, interacts with NIX and enters the mitochondrial membrane space via Tom40 and Tom70. TR3 interacts directly with ANT-1 and promotes VDAC1 action to alter the MMP and thus induce mitophagy. ⑤ Rubicon and Beclin-1 bind directly to Na^+^-K^+^-ATPase to promote autosis. This graph was drawn with Figdraw.

AT-101, a natural compound from cotton seeds, induced excessive mitophagy in glioma cells by upregulating the expression of heme oxygenase 1 (HMOX1) and the mitophagy receptors BNIP3 and BNIP3L [61]. 1-(3,4,5-Trihydroxyphenyl) nonan-1-one (THPN) drives excessive mitophagy in melanoma. The nuclear receptor TR3 (Nur77 or NGFI-B) is translocated to the outer mitochondrial membrane (OMM), interacts with NIX, and enters the mitochondrial space through the channel proteins Tom40 and Tom70, promoting alteration of the mitochondrial membrane potential by permeability transition pore complex ANT1-VDAC1 and induction of excessive mitophagy (Fig. 3) [62]. Akt2 phosphorylation of TR3 inhibits its translocation and reduces the therapeutic sensitivity of cancer cells to THPN [63].

In recent years, sphingolipids have been identified as key molecules in the induction of lethal mitophagy [64, 65]. Dasari et al. treated A549 non-small cell lung cancer (NSCLC) cells with resveratrol and identified GBA1 as a “switch” that induces ADCD, as discovered by short hairpin RNA (shRNA) survival screening [65]. GCase catalyses the conversion of glucose ceramide to glucose and lysosomal ceramide, the latter of which can be reconverted to ceramide in the ER after lysosomal breakdown. Under stress conditions, the mitochondrial fission molecule Dynamin-related protein 1 (Drp1) is nitrosylated at C644, releasing p17/PERMIT. p17/PERMIT moves near the ER and binds to CerS1 in the ER, facilitating CerS1-induced ceramide-dependent mitophagy via the OMM [64, 66]. In resveratrol-treated A549 cells, knockdown of GCase significantly inhibited ADCD, and its overexpression upregulated C16:0, C18:0, C20:0 and C24:1 ceramide levels [65]. Furthermore, knockdown of GBA1 delays midgut degradation in Drosophila larvae, suggesting that GBA1-mediated ADCD is a conserved mechanism [67]. In addition, sphingolipids affect the stability of autophagolysosomes. Δ9-Tetrahydrocannabinol (THC) inhibits the translocation of dihydroceramide from the ER to the Golgi apparatus, and accumulated dihydroceramide is enriched in autophagosomes to promote LMP [68]. Notably, in contrast to loperamide, which causes impaired lysosomal degradation leading to sphingolipid accumulation [47], the small-molecule anticancer drug ABTL0812 reduces DEGS1 activity to increase the level of dihydroceramide, which mediates cytotoxic autophagy via the activation of ER stress/ the UPR [69]. Studies have shown that Drp1-ceramide activation induces excessive mitophagy. Thomas et al. found that HPV-E7 promoted ceramide-induced mitophagy and alleviated head and neck squamous cell carcinoma development by inhibiting RB activity to activate Drp1 [70]. Inhibition of FLT3-ITD has been shown to promote cell death through Drp1 activation of CerS1/C18 ceramide synthesis in acute myeloid leukemia samples [71].

Alterations in lipid metabolism may contribute to the imbalance between protective lysophagy and lethal mitophagy or ER-phagy, and this alteration is evident in almost all types of ADCD (ER-phagy, mitophagy and normal autophagy). Notably, excessive autophagy may activate other processes to promote cell death. In conclusion, excessive degradation of organelles by either ER-phagy or mitophagy is not the only cause of cell death.

### Autosis

Autosis is a novel form of ADCD. Notably, whether autosis is truly a form of ADCD is debated, and although autosis-mediated death cannot be completely rescued after pharmacological inhibition of autophagy, we include a description of autosis as a form ADCD in this paper. Autosis has recently been identified and is characterized by an increase in the number of autophagic vacuoles and focal swelling of the perinuclear space, with cell death mainly associated with Na^+^, K^+^-ATPase activity (Fig. 3) [72]. Liu et al. named this phenomenon “autosis” after observing it while treating HeLa cells with the autophagy-specific activator Tat-Beclin1 [73]. Autosis mediates a unique mode of death that does not depend on other modes of death or on excessive “self-eating”. Research on autosis is focused on the cardiovascular system. Nah et al. found that ischemia‒reperfusion (I/R) induced myocardial cell autosis in mice, with changes in autophagic flux occurring in two phases, an early increase and a late decrease, with both phases promoting autosis [74]. Mechanistically, the transcription factor EB (Tfeb) is activated in both early and late stage autosis-related autophagic flux, and the inhibition of late autophagy is mainly mediated by Rubicon [74, 75]. Interestingly, Rubicon inhibits autophagy mainly by binding to Beclin1 [76, 77], but Beclin1 binds directly to Na^+^, K^+^-ATPase during ischemia to promote autosis [78]. Therefore, Rubicon may play other roles in autosis as a cofactor of Beclin1, and exploring the relationship among Rubicon, Beclin1 and Na^+^, K^+^-ATPase in the process of autosis will help to further elucidate the mechanism underlying autosis.

The sodium-glucose cotransporter 2 (SGLT2) inhibitor EMPA significantly alleviates myocardial ischemic injury by inhibiting excessive autophagy and autosis in cardiomyocytes through the downregulation of Na/hex exchanger 1 (NHE1) activity [79]. Activation of autophagy in cardiomyocytes under stress conditions induces both apoptosis and autosis, and crosstalk between these death pathways is evident before p62 activation [80]. Studies on autosis are still in their infancy, and the mechanism leading to cell death may be related to excessive membrane depletion, disruption of cholesterol metabolism in the ER/external nuclear membrane or altered ion transport and osmotic pressure in the plasma membrane.

The mechanisms by which drugs or compounds mediate ADCD have been summarized in Table 1.

**Table 1:** Drugs and other compounds that regulate ADCD.

Intervention Factors	Mechanism	Mode	Citation
RCD	–	Normal autophagy	[25, 27]
Bax/Bak KO	–		[26]
SH003	Activates ATF4 and inhibit G9a		[34]
Kaempferol	Activates IRE1-JNK-CHOP and inhibits G9a		[35]
Tunicamycin	Promotes ceramide synthesis through GBA1 action		[40]
Resveratrol	Activates GBA1 to promote ceramide synthesis		[65]
THC	Promotes dihydroceramide accumulation in lysosomes, which leads to LMP		[68]
ABTL0812	Promotes dihydroceramide accumulation, which activates the ATF4-DDIT3-TRIB3 pathway		[71]
Loperamide	Activates the ATF4-FAM134B axis and TEX264, leads to lipid accumulation in lysosomes and lipid ROS production	ER-phagy	[36, 47]
Z36	Activates FAM134B and the UPR		[37]
Sorafenib	Activates FAM134B		[38]
Activate smARF	smARF bind to parkin to promote mitophagy	Mitophagy	[58–60]
AT-101	Activate BNIP3, BNIP3L and HMOX1		[61]
THPN	TR3 translocation to mitochondrial gap change membrane potential		[62]
C_18_-Pyr-Cer	Activate CerS1/C_18_-Drp1		[64]
HPV	HPV E7-E2F5-Drp1-ceramide		[69]
Inhibition of FLT3-ITD	Activate Drp1-ceramide		[70]
Tat-Beclin	Activates Na^+^-K^+^ATPase	Autosis	[72]
Ischemia/Reperfusion	Activates Tfeb and inhibits Rubicon		[74, 75]
Tat-Beclin	Beclin1 binds to Na + -K + ATPase		[78]
Homocysteine and copper	Activates p22^phox^ and NOX-mediated p62 upregulation		[80]

## Autophagy-mediated cell death

### Apoptosis

Apoptosis is classified as intrinsic or extrinsic apoptosis based on the mechanism that triggers it [24].

Intrinsic apoptosis is activated by various stress conditions and is mediated mainly by the anti-apoptotic protein Bcl-2 and the proapoptotic proteins Bax and Bak. Extrinsic apoptosis is mediated by membrane death receptors such as Fas cell surface death receptor (FAS/CD95/APO-1) and TNF receptor superfamily member 1 A (TNFRSF1A), which are activated by binding to their cognate ligands. Both pathways lead to the release of cytochrome c, triggering a cascade of caspase signaling [24].

Autophagy and apoptosis are critical processes that regulate cell survival and death, and the interactions involved in apoptosis and autophagy differ in different biological contexts. The following three theories describe autophagy-mediated apoptosis: 1) autophagy inhibition of apoptosis; 2) autophagy promotion of apoptosis; and 3) autophagy is merely coactivated with apoptosis, and there is no interaction between the two pathways.

**Fig. 4:**
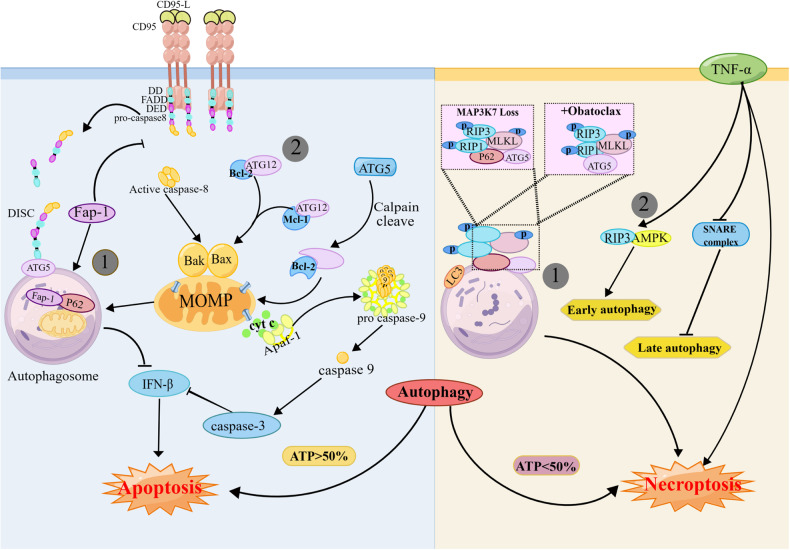
The relationship between autophagy and apoptosis or necroptosis. Autophagy mediates apoptosis and necroptosis in two main ways. ① Both forms of RCD rely on the dynamic functioning of autophagy. Autophagosomes engulf mitochondria and Fap-1 or function as platforms for the assembly of necrosis complexes, which promote cell death. ② Autophagy molecules bind to apoptotic or necrotic molecules to promote cell death. During apoptosis, ATG5 and ATG12 bind to Bcl-2 and Mcl-1 to promote apoptosis. In necroptosis, RIPK3 activates early autophagy via AMPK. This graph was drawn with Figdraw.

The following two main mechanisms explain autophagy-mediated apoptosis: **①** Autophagy leads to cell death through the phagocytosis of apoptotic molecules or organelles. The tyrosine phosphatase Fap-1 is a negative regulator of Fas (Fig. 4). In Fas-induced apoptosis, p62 recognizes and binds to Fap-1, leading to its specific degradation, increasing Fas phosphorylation levels and enhancing cell sensitivity to apoptosis [81]. Autophagosomes in BAX-/BAK-mediated apoptosis inhibit IFN-γ secretion by phagocytosing mitochondria, ensuring immune-silencing during apoptosis [82]. In SKI-1-treated cells, FADD is translocated to ATG5-ATG12-positive autophagosomal membranes to form a complex that continuously recruits and activates Caspase8 to promote apoptosis [25]. ② Autophagy molecules promote apoptosis by directly binding to apoptotic molecules. During autophagy, ATG12-ATG5 coupling plays a key role in the lipidation of LC3. Interestingly, both ATG12 and ATG5 promote apoptosis and exert anticancer effects independent of each other. Under physiological conditions, free ATG12 is degraded in a proteasome-dependent manner [83]. Following cell treatment with proteasome inhibitors, stably expressed ATG12 binds to and inactivates the antiapoptotic proteins Bcl-2 and Mcl-1 via a BH3-like motif, resulting in BAX activation, mitochondrial release of cytochrome c, and enhanced chemosensitivity [84]. ATG5-induced apoptosis is associated with calpain, which cleaves ATG5 to produce a short amino-terminal truncated fragment. Truncated ATG5 is translocated to mitochondria to block Bcl-2 and Mcl-1, altering the degree of mitochondrial outer membrane permeabilization (MOMP) and causing a cascade of activation of caspases [85].

### Necroptosis

Necroptosis is an inflammatory form of RCD that is induced by death receptors such as TNFRSF1A/TNFR1. When Caspase8 is inhibited, phosphorylation of receptor-interacting protein kinase 1 (RIPK1) and receptor-interacting protein kinase 3 (RIPK3) activates the downstream protein mixed lineage kinase domain-like protein (MLKL), driving its translocation to the plasma membrane and causing cell content leakage [24, 86]. Additionally, because of the partial overlap in the mechanisms underlying necroptosis and apoptosis, the machinery can be interchanged in some cases.

This mode of cell death is not static, and autophagy can switch from mediating apoptosis to necroptosis in specific cellular environments. ATP is the mediator of this death mode switching and initiates necrotic apoptosis when the amount of ATP available in a cell is less than 50% [87]. The switch between necroptosis and apoptosis is achieved by increasing cellular ATP utilization through “self-eating” (when ATP reserves are greater than 50%) [88]. The autophagosome membrane, an apoptotic “signal tower”, is at the core of another apoptotic mode conversion mechanism. MAP3K7 is a tumor suppressor gene. In the absence of MAP3K7, p62 recruits and mediates the translocation of RIPK1 to the autophagosomal membrane, inducing necrosis by assembling the necrosis complex, and the death modality is converted to apoptosis when p62 is knocked out [89].

In contrast to apoptosis, autophagy-mediated necroptosis usually depends on the structure or function of the autophagosome; for example, autophagy-mediated necroptosis is mediated via the aforementioned “signal tower”. Whether autophagy and necroptosis molecules engage in crosstalk is debated, as autophagy and necroptosis are regulated by separate mechanisms after different pharmacological treatments. Treatment of rhabdomyosarcoma cells with obatoclax (GX15-070), a small-molecule inhibitor of Bcl-2, stimulates ATG5 recruitment signals at the autophagosomal membrane, which serves as a platform where RIPK1, RIPK3 and FADD assembly mediates necrosis, but knockdown of RIPK1 does not inhibit autophagy (Fig. 4) [90]. In contrast, in coxsackievirus B3-infected intestinal epithelial cells, RIPK3 positively regulates autophagy, and its deletion inhibits autophagic flux and leads to the accumulation of autophagosomes [91]. Some evidence suggests that necroptosis promotes early autophagy and inhibits late autophagy. RIPK3 binds to AMPK to trigger autophagy during TNF-induced necroptosis, but TNF disrupts the SNARE complex and inhibits autophagosome–lysosome fusion [92]. There are several questions about these modes of cell death that remain unanswered, including the following: 1. What is the relationship between autophagy and necroptosis? 2. Is the activation of autophagy mediated by necroptosis, or is it an independent process? 3. The autophagosome membrane functions as a signaling platform for the necrosis complex; does it also recruit other regulatory molecules that participate in signal transduction. Autophagy is essential for necroptosis [93], and further exploration of the crosstalk between autophagy and necroptosis may help us discover potential therapeutic approaches.

### Ferroptosis

Ferroptosis, which is a form of programmed necrosis that has been identified in recent years, is activated mainly by the accumulation of iron and the production of lipid peroxides (lipid-ROS). No indicators of autophagy were detected in early studies of ferroptosis [94]. Fortunately, autophagy has been found to play a key role in iron uptake and export, redox homeostasis, and lipid metabolism; therefore, ferroptosis has been identified as a form of ADCD [95–97].

**Fig. 5:**
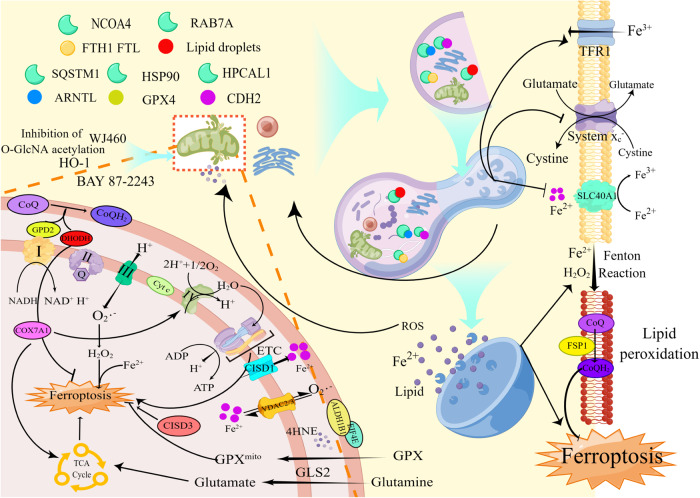
The relationship between ferroptosis and autophagy. Autophagy promotes ferroptosis by altering cellular iron content, lipid storage, redox homeostasis and energy status. During autophagy, the selective autophagy receptors NCOA4, RAB7A, and p62 bind and degrade substrates such as FTH1, lipid droplets, ARNTL, and SLC40A1. Moreover, autophagy can affect the expression of TFR1 and System X_C_^-^. The TCA cycle, ETC and the input of Fe^2+^ promote ferroptosis in mitochondria, and the input of GPX inhibits mitochondrial lipid peroxidation. DHODH1 and FSP1 are involved in inhibiting lipid peroxidation at the mitochondrial membrane and plasma membrane, respectively. This graph was drawn with Figdraw.

Selective autophagy is the main mode of autophagy-dependent ferroptosis, including (but not limited to) 1) degradation of intracellular ferritin (FTH1, FTL, and FTMT) and iron export protein (SLC40A1) via mitophagy and NCOA4 (Nuclear receptor coactivator 4)-mediated ferritinophagy [97–100]; 2) RAB7A-dependent lipophagy of lipid droplets [101]; 3) degradation of ARNTL by SQSTM1-dependent clocophagy [102]; 4) degradation of Glutathione peroxidase 4 (GPX4) by chaperone-mediated autophagy [103]; and 5) degradation of Cadherin 2 (CDH2) by Hippocalcin like 1 (HPCAL1)-mediated selective autophagy [104]. As the aforementioned factors have been previously described in excellent articles [95, 96], we will not discuss them here (Fig. 5).

The following tree main characteristics distinguish mitophagy and ferroptosis: (1) the role of mitochondria; (2) the relationship between mitophagy and ROS levels; and (3) the significance of organelle phagocytosis via autophagy.

#### The role of mitochondria in ferroptosis

Mitochondria appear to play different roles in different models of ferroptosis [105]. Energy stress effectively inhibits ferroptosis induced by cysteine deprivation or GPX4 inhibition [106]. Glutamine metabolism, the TCA cycle, and the electron transport chain (ETC) play important roles in cysteine deprivation-induced ferroptosis, suggesting that mitochondria promote ferroptosis. Notably, deletion of fumarate hydratase (FH), an enzyme involved in the TCA cycle, renders renal cancer cells resistant to ferroptosis [107]. Under these conditions, mitophagy is a negative regulator of ferroptosis in most cases. The action of CISD3, a cytochrome c oxidase subunit, is inhibited during reprogrammed leukemia cell metabolism, which is promoted via glutamine catabolism and OXPHOS. Activation of mitophagy inhibits CISD3 knockdown-induced ferroptosis [108]. COX7A1 increases the sensitivity of NSCLC cells to cysteine deprivation-induced ferroptosis by enhancing the activity of the TCA cycle and ETC complex IV [109].

In contrast, pharmacological treatment showed that mitophagy promotes ferroptosis. The mitochondrial complex I inhibitor BAY 87-2243 increased mitophagy-dependent ROS levels, leading to the ferroptosis of melanoma cells [93]. Overexpression of heme oxygenase-1 (HO-1) in BAY 11-7085-treated cells caused mitochondrial dysfunction and mitophagy, which promoted ferroptosis [110]. Treatment of breast cancer cells with mitochondrion- targeted deferoxamine (mitoDFO) impaired iron–sulfur [Fe-S] cluster/heme biogenesis and inhibited mitochondrial respiration, inducing mitophagy and promoting ferroptosis [111]. Ferroptosis was also induced by targeting myoferlin and mitochondrial iron metabolism, which promoted mitophagy [112]. However, the use of the mitophagy inhibitor Mdivi1 inhibited ROS production and restored cell viability (described in the section “The relationship between mitophagy and ROS in ferroptosis”) [112]. There are two possible reasons for the different roles of the mitochondria in ferroptosis as follows: (1) Mitochondria are metabolism hubs, and the mechanisms of ferroptosis may vary greatly under different experimental conditions; and (2) the energy metabolism and quality control in cells undergoing autophagy are coordinated, but the opposite situation is observed in ferroptosis [113]. Since autophagy recycles small molecules such as amino acids, it can theoretically inhibit ferroptosis by inducing energy stress. In contrast to this theory, it has been posited that autophagy is the main mechanism mediating ferroptosis. Activation of the autophagy initiation signal AMPK and inhibition of mTOR both mitigate the onset of ferroptosis [114, 115]; furthermore, it has been reported that the type of selective autophagy induced differs under ferroptotic and starvation conditions [116]. These results suggest that selective autophagy may be an independent mechanism that promotes ferroptosis.

#### The relationship between mitophagy and ROS in ferroptosis

Mitochondria promote cell survival by regulating processes such as energy metabolism and redox homeostasis during ferroptosis [114, 117]. Although mitophagy can trigger the onset of ferroptosis [118, 119], the relationship between ROS levels and mitophagy initiation during ferroptosis remains unclear. H_2_O_2_ (a type of ROS) and Fe^2+^ are important substrates for the Fenton reaction, but some studies have shown that ROS function as upstream signaling molecules that activate mitophagy to promote ferroptosis [93, 112, 119], which contradicts the function of mitophagy in scavenging ROS [97, 119, 120]. This contradiction may explain ROS production from the perspective of Fe^2+^. Fe^2+^ reacts with H_2_O_2_, which produces lethal free radicals that peroxidize unsaturated fatty acids [94]; therefore, cells maintain free iron at very low levels through sophisticated regulatory processes. During ferroptosis, autophagy facilitates the production of free iron by affecting the levels of iron input and output, storage, and transport [99, 100, 120]. Moreover, mitochondria function as buffers for cytoplasmic free Fe^2+^ to inhibit ferroptosis, while mitochondrial cleavage and autophagy are promoted following inhibition of O-GlcNAcylation [98]. Thus, mitochondria function as iron pools involved in redox, are capable of storing a certain amount of iron in the physiological state [121], and function as buffers to attenuate iron toxicity in cells [98]. Mitophagy may also contribute to the clearance of certain ferroptosis inhibitors, such as ALDH1B1, which inhibits ferroptosis by scavenging 4-hydroxynonenal (4HNE) [122]. After mitophagy induction, autophagolysosomes breakdown antioxidant substances and ferritin [123], allowing for the release of free Fe^2+^ and the Fenton reaction to proceed with unreduced ROS [124]. Fe^2+^ and Lipid-ROS are released from lysosomes and stimulate mitophagy, leading to a vicious cycle of iron leakage. In summary, mitophagy may be triggered by cells that help maintain balance. Compared with ROS degradation via mitophagy, increased ROS production may be promoted via the decomposition of ferroptosis inhibitors such as antioxidant substances or a decrease in the ferritin level in mitochondria. Of course, this hypothesis is applicable only to the inhibition of ferroptosis via mitochondria and cannot explain lipid peroxidation in mitochondria.

#### Autophagy and mitochondrial lipid peroxidation

Lipid peroxidation occurs not only at the plasma membrane but also at the mitochondrial membrane [125]. The relationship between autophagy and the organelles involved in lipid peroxidation is worth considering. Knocking down CDGSH iron sulfur domain 1 (CISD1) promotes mitochondrial iron accumulation and lipid peroxide production [125]. The lipid transporter protein Sterol carrier protein 2 (SCP2) transports hydroperoxide species and phospholipid hydroperoxide families to the mitochondria during ferroptosis, promoting lipid peroxidation at the mitochondrial membrane [126, 127]. In contrast, mitochondria can trigger a set of antioxidant mechanisms to counteract lipid peroxidation. Dihydroorotate dehydrogenase (DHODH) and G3P dehydrogenase 2 (GPD2) promote the conversion of mitochondrial membrane CoQ to CoQH_2_ and thus protect mitochondrial membrane integrity [128, 129]. The specific protective mechanism of the mitochondrial membrane suggests that redox homeostasis may be necessary in the organelles of other subcellular compartments, such as the ER [130], peroxisomes [131], lysosomes [132], and the Golgi apparatus [133]. Interestingly, although the prevailing theory suggests that autophagy positively regulates ferroptosis [97, 98, 100], there is no plausible explanation for the facilitation of ferroptosis via the autophagosome phagocytosis of organelles. Thus, does autophagy function as only one of the mechanisms that regulates the redox of these organelles to promote ferroptosis? Cell and organelle membranes include unsaturated fatty acids that can undergo lipid peroxidation; therefore, autophagosomal membranes derived from organelle membranes (e.g., ER and Golgi) can be subjected to lipid peroxidation. Can autophagosomes shuttling through cells function as “mobile bombs,” causing serious damage to cells? Exploring the precise regulatory mechanisms of autophagy and the roles of organelles will help us further understand the significance of autophagy in ferroptosis.

The mechanisms by which drugs or compounds mediate AMCD have been summarized in Table 2.

**Table 2:** Drugs and other compounds that regulate cellular AMCD.

Intervention Factors	Mechanism	Mode	Citation
SKI and Bortezomib	Autophagosome provides a platform for Caspase-8 recruitment and activation	Apoptosis	[23]
Homocysteine and copper	Cleavage of ATG5 to block Bcl-2		[80]
Fas Ligand	Autophagosome phagocytosis of Fap-1 Inhibits Fas		[81]
ABT-737	Autophagosomes engulf mitochondria and inhibit IFN-β secretion		[82]
MG132	ATG12 binds to and inactivates Bcl-2		[83, 84]
Calpain	Calpain cleaves ATG5 to block Bcl-2		[85]
TRAIL and MAP3K7 KO,GX15-070	Autophagosome membrane platform promotes assembly of the necrosis complex	Necroptosis	[89]
Coxsackievirus B3	Activates RIPK3		[91]
TNF-α	RIPK3 activates AMPK to promote early autophagy and inhibit late autophagy		[92]
BAY 87-2243	Promotes ROS-dependent mitophagy		[93]
Erastin	Autophagosomes degrade FTH1, FTL, FTMT	Ferroptosis	[96–98]
	Autophagosomes degradation SLC40A1		[99]
	Autophagosomes degrade lipid droplets through RAB7A action		[100]
	Autophagosomes degrade ARNTL through the action of SQSTM1		[101]
	Partner-mediated autophagosome degradation of GPX4		[102]
	Autophagosomes degrades CDH2 through the action of HPCAL1		[103]
	ROS activates autophagy, upregulates TFR1 level		[116]
BAY 87-2243	Promotes ROS-dependent mitophagy		[93]
Inhibition of O-GlcNA acetylation	Induces mitophagy and iron-specific autophagy and promotes F^2+^ release		[97]
WJ460	Induces mitophagy		[111]
Celastrol and erastin	Induces autophagy and mitophagy		[115]

## Autophagy in the clinic: challenges and hope

Currently, the clinical application of autophagy mainly involves inhibiting autophagy and combining it with chemotherapeutic drugs to promote cell death in tumors, and there is no strategy to cure the disease by inducing ACD. However, this paper shows that autophagy is involved in almost all cell death modes, which indicates that targeting autophagy is a powerful strategy for curing diseases. However, it will take some time for ACD to be applied in the clinic, and we propose three questions based on the possible future development of ACD: 1) How can the functions of autophagy be identified? 2) How can an ideal autophagy-targeting drug be developed? 3) Is there a more effective clinical treatment based on the different functions of autophagy?

### How can the functions of autophagy be identified?

Autophagy exhibits dual effects in most diseases, and identifying the effects of autophagy in pathological tissues is a prerequisite for targeted autophagy-based therapy. However, the identification of autophagy effects in clinical trials is based on findings from cellular or animal experiments. Considering recent studies, we classified autophagy into the following two categories according to its mechanism of action: monophasic autophagy and biphasic autophagy. (1) Monophasic autophagy. Autophagy can promote either cell survival or death in tissues, which is the common model in most studies. We can determine the effects of autophagy by detecting protection-related (Sirt1 [134]) or lethality-related (β-Thujaplicin [135] and GBA1 [65]) markers of autophagy. However, marker sensitivity and specificity need to be evaluated, and their development for clinical use in molecular diagnostics is still in its early stages. (2) Biphasic autophagy. Although biphasic autophagy has not been systematically investigated, this model of autophagy has been reported. Loperamide induced excessive ER-phagy to cause cell death [38], while it also eliminated lysosomes during LMP through lysophagy [47], suggesting that autophagy may be involved in different processes in the same cells. In sorafenib-treated hepatocellular carcinoma (HCC) cells, autophagy was activated to inhibit apoptosis and mediate cellular chemoresistance [136], but sorafenib also induced autophagy-dependent ferroptosis in HCC cells [130]. Furthermore, the involvement of different molecules in the formation of autophagosomes induced by starvation or erastin (a ferroptosis inducer) suggested that the different roles autophagy may be mediated via different regulatory mechanisms [116]. Therefore, identifying the “tipping point” between autophagy-mediated cell protection and killing or identifying and quantifying the expression of marker molecules for different autophagy-related functions to identify which autophagy functions dominate cell survival may be a strategy to determine the predominant role of autophagy under different conditions.

### How can ideal autophagy-targeting drugs be developed?

Current therapeutic strategies are mainly based on autophagy inhibition and are limited to tumors; one of the main reasons for this phenomenon is the lack of clinically applicable autophagy activators that have been developed. Theoretically, the following two clinical outcomes are based on lethal autophagy: (1) The promotion of autophagy to kill tumors, bacteria or viruses, and (2) the inhibition of autophagy to protect tissues and cells. However, lethal autophagy in cells under physiological or pathological conditions is rarely triggered, and it is not practical to treat diseases by inhibiting lethal autophagy in clinical applications. Furthermore, the induction of lethal autophagy to destroy tumors and pathogens is only one of the therapeutic purposes of autophagy activation; therefore, we add to the scope of this discussion by suggesting the strategy of promoting autophagy to treat disease. Previous studies have used rapamycin, metformin, carbamazepine, cardiac glycosides, and statins to promote autophagy for the treatment of disease [137]. However, these drugs act mainly on the upstream signaling of autophagy pathways and enhance autophagy while also playing a role in other pathways, such as apoptosis, the immune response and cell growth, which can lead to side effects. Therefore, the development of selective activators of autophagy is more promising than a treatment based on the pan-active activation of autophagy.

#### Beclin-1

Activation of Beclin-1 promotes autophagy initiation and autophagosome formation, and it has shown remarkable therapeutic potential in tumors, Parkinson’s disease (PD), and the induction of anti-pathogenic microbial responses. Initially, Levine et al. developed a peptide derived from Beclin-1, named Tat-beclin, in a study on pathogenic microorganisms, and it enhanced autophagy to effectively prevent chikungunya virus and West Nile virus infection in neonatal mice [73]. Since its discovery, Tat-beclin has been shown to mediate autosis [72]. Based on the study of Levine’s group, Zhang et al. encapsulated Tat-beclin in biodegradable lipid-coated hybrid lactic-co-glycolic acid (PLGA) nanoparticles to selectively induce autosis in HIV-infected CD4^+^ T cells and macrophages; notably, the uninfected cells were largely unaffected, suggesting that Tat-beclin shows great potential as an antiviral therapy [138, 139]. Wang et al. developed the following novel antitumor strategy based on the idea of “expanding revenue sources and reducing expenses”: the activation of early autophagy using Tat-beclin and the inhibition of late autophagy using hydroxychloroquine (HCQ) to induce excess autophagosome production in tumor cells [140]. Xu et al. found that knockdown of DUSP4, an upstream signaling protein involved in the Beclin-1-Bcl-2 interaction, attenuated the relationship between Beclin-1 and Bcl-2 and promoted apoptosis [141]. Furthermore, induction of Beclin-1-dependent autophagy in the context of sepsis conferred significant protection against LPS damage and reduced inflammation in mouse hearts [142].

#### Autophagy receptors

Targeting autophagy receptors is an emerging strategy for the clinical regulation of autophagy-selective degradation. p62 is a classical autophagy receptor that recognizes cargo and delivers it to autophagosomes. Dehydroepiandrosterone mediates p62 expression to promote ACD in HCC cells. Additionally, p62-dependent ACD is particularly effective in cells that are resistant to apoptosis [143]. Ginkgolide targets p62 to induce ACD and promote oxidative damage in NSCLC [144].

NCOA4 is an autophagy receptor for ferritinophagy. Caryophyllene oxide is a targeted regulator of NCOA4, which facilitates the interaction of NCOA4 with FTH1 and the delivery of FTH1 to lysosomes to promote ferroptosis [145]. Combined treatment with d-borneol and cisplatin promotes NCOA4-mediated ferritinophagy while upregulating PRNP expression and downregulating PCBP2 expression to promote ferroptosis in HCC cells [146]. However, increased NCOA4-mediated ferritinophagy has been shown to help maintain the growth of pancreatic cancer tumors by promoting mitochondrial iron–sulfur cluster protein synthesis and mitochondrial respiration; these different outcomes may be due to different degrees of NCOA4 activation [147]. In summary, the selection of the most appropriate dose of an NCOA4 activator to kill tumors while minimizing damage to surrounding tissues is a major challenge.

HPCAL1 is a neuronal calcium sensor that has recently been identified as an autophagy receptor that induces ferroptosis. HPCAL1 mediates the degradation of CDH2 and reduces plasma membrane tension to promote lipid peroxidation. Inhibition of HPCAL1 inhibited pancreatic cancer progression and pancreatitis development in a mouse model [104], suggesting that targeted enhancement of HPCAL1 expression may be a potential antitumor strategy.

#### Gcase

As previously mentioned, the gene encoding GCase, GBA1, is a positive mediator of ADCD [65], and the ability to mediate ADCD is highly conserved among organisms [67]. Clearly, GCase is the preferred target for the development of drugs targeting ADCD pathways in tumor therapy. In addition, enhanced GCase expression stimulates the lysosomal degradation of α-synuclein. In the postmortem brains of PD patients, approximately one-half of the GCase identified was located on the lysosomal surface, and this mis-localization was attributed to a pentapeptide motif mutation in GCase. This mutation inhibited chaperone-mediated autophagy, which ultimately led to the accumulation of α-synuclein [148]. As GCase does not cross the blood‒brain barrier, the development of small-molecule chaperones to correct GCase folding and thus enhance GCase activity and lysosomal function is a feasible strategy [149]. Small-molecule chaperones specifically target misfolded GCases trapped in the ER, stabilize its active form and increase the amount of GCase transported to lysosomes [150]. Several small-molecule chaperones, such as amiloride [151], AT2101 (isofagomine) [152] and histone deacetylase inhibitors [153], have been shown to increase GCase activity and reduce oxidative stress in PD fibroblasts carrying GBA1 mutations or enhance GCase activity in GBA macrophage models. In addition, through high-throughput screening, a pyrazolopyrimidine derivative that did not inhibit GCase but contributed to its translocation to lysosomes was identified, and it showed promising effects in preclinical experiments [154].

#### Other compounds

MTMR is a myotubular protein-related phosphatase that antagonizes the formation of autophagosome membrane structures. Autophagy enhancer-67 (AUTEN-67) and AUTEN-99, small-molecule inhibitors of MTMR, significantly increased autophagic flux in both in vitro and in vivo models. AUTEN-67 promoted longevity and protected neurons from stress-induced cell death and caused no severe side effects [155]. AUTEN-99 blocked the progression of neurodegenerative symptoms in Drosophila models of PD and Huntington’s disease [156]. Notably, both of these compounds slowed the ageing of Drosophila rhabdomyocytes [157].

TFEB coordinates lysosomal biogenesis and is a key link between the upstream signaling pathways regulating macroautophagy and lysosomes. 2-Hydroxypropyl-β-cyclodextrin chemically activated TFEB to promote autophagy-based clearance of α-syn [158, 159]. Arsenic trioxide and dihydromyricetin activated TFEB to induce ADCD in cancer cells [160, 161].

Although small molecules or compounds targeting autophagy have been rapidly developed, the development of these potential treatments from the preclinical stage to the clinical stage has been met with some resistance. (1) Autophagy-independent functions of autophagy machinery. The vast majority of autophagy-regulated molecules show autophagy-independent functions, although for some of the proteins known to be involved in autophagy, there have not been annotated non-autophagy roles (e.g., ATG2), most likely because of theoretical or technical limitations [162]. The unclear effects on non-autophagy functions are the reason that small-molecule drugs targeting autophagy molecules are not widely used in the clinic—for example, we cannot identify the other effects of small molecules involved in autophagosome formation in different patients. Two reasons for the failure to identify the autophagy-independent function of small molecules are clear. First, small molecules involved in multiple cellular functions are present in low numbers, and when one of the functions it mediates is inhibited, another function may be overactivated. For example, UVRAG interacts with an ER-tether to control COPI transport from the Golgi to the ER. However, when autophagy is activated, UVRAG dissociates from the tether and participates in autophagosome formation [163]. Second, autophagy is a redundant process that can occur independently of classical regulatory molecules; for example, degenerative autophagy, secretory autophagy and recycling autophagy can be mediated simultaneously [164]. In summary, the search for molecules that stably regulate autophagy mechanisms is a prerequisite for the development of autophagy-targeted drugs. (2) Off-target effects. Metformin, an AMPK-specific agonist, induces autophagy but also induces other signaling pathways, such as the Hedgehog [165] and ROS/JNK [166] signaling pathways, leading to off-target effects. Although wortmannin is an efficient pan-PI3K inhibitor with relatively satisfactory potency, its low selectivity for other kinases limits the expansion of its applications [167]. (3) Low efficacy Although the autophagy inducers rapamycin and alginate and the autophagy inhibitor 3-methyladenine (3-MA) have been widely used in animal or cellular disease models (to treat liver diseases, neurodegenerative diseases, etc.) and have shown clear efficacy, they are not used in the clinic because of the high concentrations needed to induce their function [168]. This limitation is similar to that of the two autophagy inhibitors currently applied in the clinic, chloroquine (CQ) and HCQ—the clinical trials based on these drugs targeting autophagy have been largely limited to cancer therapy, again due to the high concentrations required for their effectiveness [169, 170]. In summary, the following two types of problems have slowed the development of small molecules that target the autophagy pathway: methods to identify the targets of the compounds and challenges in increasing the efficacy of the compounds. The molecules involved in autophagy regulation alone are not well characterized, and therefore, the development of compounds that target multiple autophagy-regulating molecules with high potency and specificity is a potential approach to overcome these challenges.

### Is there a more effective clinical treatment based on the different functions of autophagy?

The functions of autophagy are highly dependent on the biological context, which can play different roles at different autophagy stages even in the same disease. Therefore, modulating the functions of autophagy in disease states, such as switching protective autophagy to lethal autophagy in tumors or switching lethal autophagy to protective autophagy in osteoarthritis (OA), senescence or the cardiovascular system, may considerably improve therapeutic efficacy. We think that these “switches” are widespread in diseases, but the current research on these “switches” is extremely limited, and in the following, we will focus on the transformation of autophagy in cancer and senescence, while the rest are presented in Table 3.

**Table 3:** Switches that mediate the functional transitions of autophagy.

Disease/Mold	Switches	Conversion of autophagy function	Citation
Insulin withdrawal	VCP, calpain 1 and calpain 2	ACD was induced by insulin withdrawal. The overexpression of VCP, calpain 1 and calpain 2 converted the cell death pattern to apoptosis	[26, 176]
I/R	–	Treatment with 3-MA for 20 min before hypoxic ischemia induction inhibited autophagy and increased the neuronal death rate. However, when 3-MA was used 4 hours after ischemia, it significantly reduced lesion formation.	[183]
I/R	AMPK	Ischemia induced AMPK activation and protective autophagy in the myocardium. Lethal autophagy was activated by Beclin1-dependent and AMPK-independent autophagy during the reperfusion phase	[184]
Amyotrophic lateral sclerosis	–	Autophagy defects in motor neurons caused denervation of the tibialis anterior muscle and episodes of hindlimb tremor in the early stages of the disease. However, the autophagy defect alleviated glial inflammation and blocked the activation of interneuron c-Jun at the late stage, extending the lifespan of the mice.	[185]
Virus infection	–	Autophagy in tick-borne encephalitis virus-infected macrophages was altered with an increasing duration of infection. In the early stages of infection, autophagy inhibited viral replication. In contrast, autophagy inhibited antiviral responses by limiting IFN-β production in the later stages of infection.	[186]
Rheumatoid arthritis	–	Osteoarthritic synovial fibroblasts were treated with an ER stress inducer and a proteasome inhibitor to activate autophagy. The former induced autophagy-mediated nonapoptotic cell death, whereas the latter induced protective autophagy to inhibit apoptosis.	[187]
OA	SIRT1	Autophagy selectively cleared SIRT1 to increase LOX-1 expression, leading to chondrocyte death. In contrast, under physiological conditions, SIRT1 is normally expressed induces autophagy.	[188]

#### Cancer

In tumor cells, a new set of survival mechanisms are initiated to counteract different treatment strategies. A phase II clinical trial conducted in 2013–2017 to evaluate the pharmacological treatment of advanced pancreatic ductal carcinoma showed that the autophagy inhibitor HCQ in combination with paclitaxel or gemcitabine resulted in significantly higher overall remission rates in patients [170], but more recent studies have suggested that cancer cells have evolved macropinocytosis (MP) to target and inhibit protective autophagy therapies [171]. Therefore, the need to adapt research and therapeutic strategies for autophagy treatment is urgent. Autophagy plays different roles at different stages of tumor formation, suggesting that targeting one or more key molecules may drive a switch in autophagy function. For example, in HCC cells, autophagy undergoes two transitions at different stages of hepatocarcinogenesis. First, autophagy inhibition leads to attenuated liver injury and hepatomegaly but does not affect liver fibrosis. Subsequently, liver fibrosis gradually develops into early-stage tumors, and the role of autophagy shifts from promoting liver injury to inhibiting early-stage tumor formation [172]. Finally, in the late stages of tumor development, autophagy shifts from inhibiting early tumor formation to promoting the proliferation of advanced tumors [173]. In addition, similar autophagy role switching has been identified in the development of lung cancer [174]. Some of the “switches” that mediate the transformation of autophagy function have been identified. In pancreatic cancer driven by the KrasG12D mutation, overexpression of P53 and inhibition of autophagy attenuated tumor development. However, the knockout of P53 and inhibition of autophagy promoted glucose uptake and enhanced the glycolytic and pentose phosphate pathways to induce pancreatic carcinogenesis [7]. Valproic acid (VPA) treatment promoted H4K16ac and led to the death of HeLa cells mediated through rapamycin-activated autophagy. In untreated cells in this study, rapamycin-induced autophagy failed to induce cell death [175].

Additionally, it is difficult to eradicate drug-resistant and residual cancer cells, mainly because a patient’s long-term single mode of treatment causes the cancer cells to evolve survival mechanisms. Using autophagy-based therapy, it may be possible to flexibly target key molecules at different times during tumor treatment and switch cell death patterns to increase sensitivity to treatment. Recent studies have successfully implemented examples of these therapies, such as MAP3K7 and p62, which mediated the switch between necroptosis and apoptosis (described in the section “Necroptosis”) [89]. VCP [26] and calpain [176], which mediated the switch between ADCD and apoptosis.

#### Senescence

Basal autophagy rates delay cellular senescence, mainly by removing damaged mitochondria to reduce ROS production and maintain the stemness of satellite cells, muscle stem cells, etc. [177]. Moderate overexpression of Drosophila ULK1 prolonged lifespan, but ULK1 overexpression inhibited mitochondrial metabolism and led to progressive lipid damage and shortened lifespan [178]. Narita et al. identified a specific autophagy process during H-RasV12-induced senescence, called the TOR-autophagy spatial coupling compartment (TASCC), which produced many cells that acquired the senescence-associated secretory phenotype (SASP), which promotes senescence [179]. The SASP is one of the most important causes of cellular senescence, as the secreted factors are capable of affecting not only the cell itself in an autocrine manner but also nearby cells and the tissue microenvironment. In the TASCC, autophagy degrades proteins into small molecules, such as amino acids, and these amino acids activate mTOR to induce the synthesis of various cytokines, chemokines, etc. This catabolic-anabolic coupling mechanism greatly enhances SASP factor secretion and promotes cellular senescence [180]. In addition, RAS mediates the binding of LC3 to the nuclear protein LaminB1, and some chromatin is transported with LaminB1 and degraded in the lysosome, thereby promoting cellular senescence. However, in starvation-induced autophagy, the interaction of LC3 with LaminB1 does not mediate the degradation of chromatin or LaminB1 [181]. These studies suggest that RAS may be critical in determining the different types of autophagy that are initiated. Jiang et al. found that under physiological conditions, GATA4 binds to the autophagy receptor p62 and is degraded by selective autophagy. However, selective autophagy of GATA4 was disabled in the presence of senescence-inducing stimuli, and GATA4 became stable, activating the SASP program and inducing cellular senescence [182].

## Conclusions

Autophagy with dual effects is similar to a maze with two exits, and the bifurcation points of the two pathways are becoming clearer. Protective and lethal autophagy may be triggered simultaneously, but one type of autophagy predominates in different stages of disease development or under intervening conditions [47]. AMCD and ADCD are both forms of RCD, and a finely tuned system of regulation mediates these processes. Alterations in the expression or posttranslational modifications of specific molecules can determine the autophagy-mediated transition between the protection and killing of cells, but the exact mechanisms of action and the upstream molecules activating this transition remain unclear. The study and application of autophagy remain limited. For experiments, effective experimental methods to analyze the dynamic switching process are lacking, which is especially important for in vivo experiments. No specific inhibitors or activators of autophagy are available in the clinic, and the efficacy and pharmacological safety of clinical drug candidates that trigger autophagy need to be investigated further.

Investigation should not be limited to a single function of autophagy (e.g., protective, lethal, or mediating a single mode of death) but should focus on the flexibility of autophagy in regulating cell death and explore the mechanisms underlying autophagy switching in tumors. Exploring autophagy-related death is a priority for the induction of autophagy transitions. Therefore, the discovery of marker molecules of autophagy-associated death and related metabolic mechanisms, the exploration of clinically applicable target drugs, sensitive indicators of autophagic flux and autophagy outcomes, and the application of these markers in combination with autophagy transitioning in the clinic may transform disease treatment.

## Data Availability

All data generated or analyzed during this study are included in this published article.
